# Epidemiological report and diagnostic approach used in the neuromuscular population of Liege, Belgium

**DOI:** 10.1186/s13023-025-03963-2

**Published:** 2025-08-29

**Authors:** Charlotte Mouraux, Tamara Dangouloff, Margaux Poleur, Laurane Mackels, Laura Vanden Brande, Aurore Daron, Laurent Servais, Alain Maertens de Noordhout, Stéphanie Delstanche

**Affiliations:** 1 Clinical Genetics Department, CHU Liège, 1 Avenue de L’Hôpital, 4000 Liège, Belgium; 2 Neuromuscular Reference Center, Department of Pediatrics, University Hospital Liège, Liège, Belgium; 3 University Department of Neurology, CHR Citadelle, Liège, Belgium; 4 University Department of Pediatrics, CHR Citadelle, Liège, Belgium; 5 MDUK Oxford Neuromuscular Centre & NIHR Oxford Biomedical Research Centre, University of Oxford, Oxford, UK; 6 Rare Movement Disorders Research Group - GIGA Human Imaging, University of Liège, Liège, Belgium

**Keywords:** Diagnostic guidelines, Genetic testing, Whole exome sequencing, Neuromuscular disorders, Prevalence, Neuromuscular Reference Center, Belgium

## Abstract

**Background:**

Patients with neuromuscular diseases (NMD) have undergone considerable technological progress in terms of diagnosis and treatment over the past few years. Specifically, next-generation sequencing (NGS) has significantly expanded genetic diagnosis. Despite this, some patients remain undiagnosed and therefore without access to specific treatments. Analyses of epidemiology and diagnostic approaches in reference centers are required to determine effective strategies to improve diagnostic rates.

**Methods:**

We studied the proportion of each NMD and associated investigations in the patient population of the Neuromuscular Reference Center (NMRC) of Liege, Belgium, in 2023. The investigation tools used included laboratory testing, muscle biopsy, muscle imaging, single-gene sequencing, targeted NGS panels, and whole-exome sequencing (WES).

**Results:**

Of the 1084 patients who were regularly followed up, more than one-third had neuropathies (36.6%) that were divided equally between genetic and acquired causes. The second most common disorder was muscular dystrophies, which represented more than a quarter (27.5%). Third, 11.2% of the patients had motor neuron diseases. The other NMD (i.e., myopathies, ataxias, spastic paraplegias, and channelopathies) ranged from 2.1% to 6. %. A total of 13.7% of the patients had unconfirmed diagnoses, 31.5% had confirmed acquired disorders, and 54.9% had genetically confirmed disorders. Among the genetic diagnoses, 32.7% were obtained by NGS. The remaining 67.3% were determined using other genetic testing methods [i.e., array comparative genomic hybridization (aCGH), multiplex ligation-dependent probe amplification (MLPA), polymerase chain reaction (PCR), southern blotting (SB)].

**Conclusion:**

More than two-thirds of patients received a definitive diagnosis without the use of next-generation sequencing. Although innovative technologies such as whole genome sequencing and long-read sequencing are expected to eventually replace NGS panels and traditional methods (e.g., MLPA, PCR, aCGH), their current cost and the complexity of variant interpretation limit their widespread use in routine clinical practice. As a result, these older techniques remain relevant and valuable in current diagnostic workflow.

**Supplementary Information:**

The online version contains supplementary material available at 10.1186/s13023-025-03963-2.

## Introduction

Neuromuscular diseases (NMD) constitute a complex group of heterogeneous disorders affecting motoneurons, muscles, nerves, or neuromuscular junctions. They are either acquired or inherited [1]. The global prevalence of NMD is approximately 170 per 100,000 people [2, 3]. Diagnostic yield is constantly increasing due to improvements in diagnostic techniques, especially in genetic testing [4]. NMD diagnosis typically involves neurological examinations, anatomopathological examination of tissue biopsies (e.g., muscle or nerve biopsies), electrophysiological studies, and genetic testing [5]. Next-generation sequencing has enabled the discovery of novel disease genes, with more than 500 genes associated with NMD to date [6]. Nevertheless, despite technological progress, around 50% of patients remain undiagnosed [7]

Regardless of etiology, affected patients frequently need multidisciplinary care due to the multisystemic impacts of NMD, such as respiratory disturbance, osteoarticular complications, or associated cardiomyopathy [8]. Some curative treatments are available for acquired disorders, such as plasma exchange, intravenous immunoglobulins (IVIg), or other immune treatments for chronic inflammatory demyelinating polyradiculopathy (CIDP) or other immune-mediated NMDs [9]. There are also treatments for genetic disorders such as nusinersen (Spinraza^®^), onasemnogene abeparvovec (Zolgensma^®^), or risdiplam (Evrysdi^®^) for spinal muscular atrophy (SMA) [10]. The use of such therapies will continue to increase according to clinical trials in progress (approximately 10,339 studies listed on clinicaltrials.org on September 4, 2023). However, these novel therapies require a precise diagnosis—and for genetic NMD, a biomolecular confirmation—in order to access treatment or clinical trials. Therefore, increasing the diagnostic yield in NMD has become essential. Many NMD diagnostic guidelines have been recently published [11–15]. However, these recommendations must be confronted with routine medical practice, which does not always allow their strict application due to a lack of resources or the complexity of clinical presentation. In particular, the use of whole genome sequencing (WGS) as a second or even first line, as recommended by these guidelines, is far from being applicable even in industrialized countries, due to its cost and complexity of interpretation. Diagnostic processes will vary due to both local epidemiology and resource availability. Therefore, the aim of this study is first to explore the local epidemiology of NMD in Belgium based on a cohort of more than a thousand patients. The second aim is to develop practical diagnostic guidelines based on the analysis of the diagnostic process in this cohort, in comparison with a literature review of the current guidelines.

## Methods

### Population

This cross-sectional monocentric epidemiological study was conducted at the Neuromuscular Reference Center (NMRC) of Liege Belgium, in 2023 and included all patients registered on July 10, 2023. The exclusion criteria were personal rejection or transient follow-up for non-chronic diseases. Foreign patients with no Belgian Social Security affiliation were also excluded.

The NMRC of Liege is one of the seven NMRC in Belgium and the only one in Wallonia (southern Belgium, excluding Brussels). It encompasses patients from Liege, Namur, Luxembourg, and Hainaut provinces, which include approximately 4,000,000 people and more than a thousand patients. Patients are followed up at the NMRC of their choice. Consequently, our data may not accurately reflect the true prevalence of the disease in the general population, although no recruitment bias is anticipated.

This project was approved by the Ethics Committee of the Citadelle Hospital of Liege on Oct 19, 2023, with the reference number “JL/rc/2093”. Written informed consent was not necessary for this cross-sectional noninterventional study.

### Data collection and classification

NMRC of Liege benefits from easy access to magnetic resonance imaging (MRI), muscle biopsy and electrophysiological explorations. Available genetic testing includes array comparative genomic hybridization (aCGH) for quantitative genome analysis; multiplex ligation-dependent probe amplification (MLPA) for recurrent copy number variant (CNV) (e.g., *DMD* gene deletion, *PMP22* gene deletion and duplication, *SMN1* exon 7 deletion); southern blot for detection of the contraction of the D4Z4 microsatellite repeat in the 4q35 region associated with facio-scapulo-humeral muscular dystrophy type 1 (FSHD1); quantitative polymerase chain reaction (qPCR) for exon 7 deletion of the *SMN1* gene; and PCR for repeat expansion (e.g., *DMPK*, *FXN*, *C9ORF72*). Regarding next-generation sequencing (NGS) technologies, filtered exome sequencing with a targeted gene panel is used for diagnosing myopathies, neuropathies, or congenital myasthenic syndromes. Genes associated with amyotrophic lateral sclerosis (ALS), such as *SOD1*, *TARDBP*, and *FUS*, are sequenced using targeted capture methods. Whole exome sequencing (WES) is prescribed in selected cases, as it remains costly and its interpretation is complex. WGS is only available for research purposes, as it is not yet reimbursed in Belgian for clinical use. Consequently, no patients underwent WGS in this observational study.

Demographic and clinical data were extracted from medical records comprising neuromuscular specialist reports, biomolecular testing, and paraclinical examinations [e.g., CPK rate, muscle MRI, muscle biopsy, electroneuromyography (ENMG)]. For genetic diseases, molecular biology confirmation was used to match the clinical presentation. For acquired diseases, international diagnostic criteria were used. For example, for CIDP, we used criteria proposed by the European Academy of Neurology/Peripheral Nerve Society (EANS/PNS) [9].

The diagnosis status was also recorded (i.e., unconfirmed, confirmed, or ongoing). Unconfirmed diagnosis indicated clinical suspicion without any paraclinical test confirmation, such as genetic confirmation for genetic disorders, muscular biopsy for myopathies, or antibodies for immunological diseases.

The NMD classification was partly based on the 2022 version of the gene table of neuromuscular disorders [16] and on the Orphanet classification of rare diseases (https://www.orpha.net). We chose to classify neuromuscular diseases into ten categories (i.e., acquired peripheral neuropathy, genetic peripheral neuropathy, inflammatory myopathy, metabolic myopathy, nondystrophic myopathy, muscular dystrophy, motor neuron disease, neuromuscular junction disease, muscular channelopathy, hereditary ataxia and spastic paraplegia). Ataxias and spastic paraplegias are classified either as movement disorders [17] or as neuromuscular disorders [16] depending on the authors. In the Neuromuscular gene table of Cohen et al. [16]—and its recent update [6]—they considered ataxias and spastic paraplegias as neuromuscular diseases. Since we used this table for classification, and because patients with ataxia and spastic paraplegia fall under the neuromuscular convention for healthcare reimbursement in Belgium, we included this group in our study.

### Analysis

Patient data were pseudoanonymized, and only sex, age, diagnosis, NMD category, and molecular confirmation data were retained for analysis. Descriptive statistics were used to characterize the study population. Frequencies (percentages) are reported for categorical variables such as NMD categories and subgroups.

Analysis and graphs were performed using RStudio version 2023.03.0 + 386 (RStudio Team (2023). RStudio: Integrated Development for R, an interface of the R program (R Core Team (2023). R: A Language and Environment for Statistical Computing_. R Foundation for Statistical Computing, Vienna, Austria).

## Results

### Population distribution

A total of 1,084 patients – including 173 children (i.e., 0 to 18 years) – were regularly followed up at the NMRC of Liege on July 10, 2023, from whom 586 (54.1%) were males and 498 (45.9%) were females (M/F ratio 1.12). The age range was 1.2 years to 88.5 years, with a median age of 45.9 years.

**Fig. 1:**
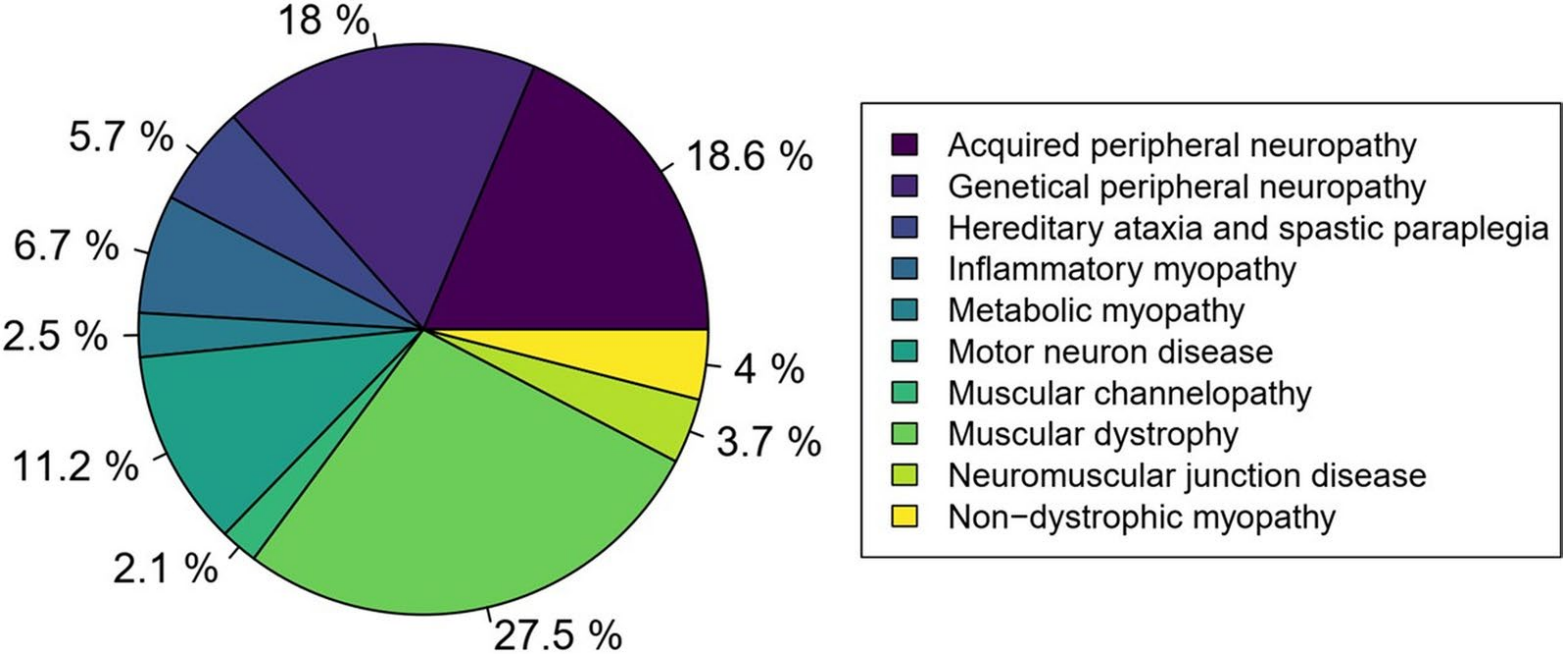
Population distribution into the ten NMD categories. N = 1,084 patients

More than one-third of patients presented with neuropathies (36.6%). They were divided equally between genetic and acquired causes. The second most common disorder was muscular dystrophies, which accounted for more than a quarter (27.5%). Third, motor neuron diseases represented 11.1% of the patients. The prevalence of other NMD (i.e., myopathies, ataxias, spastic paraplegias and channelopathies) ranged from 2.1 to 6.7% (Fig. 1).

**Fig. 2:**
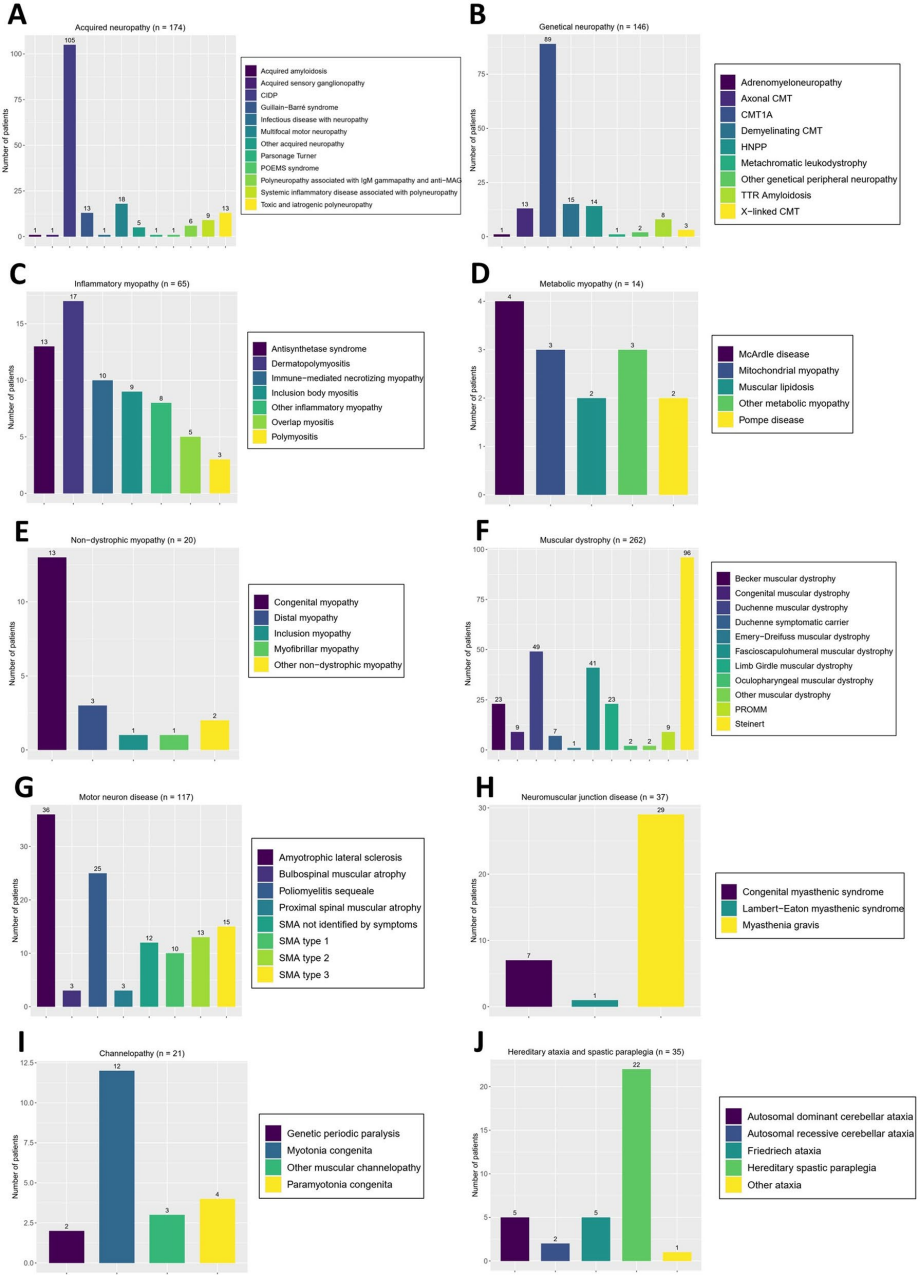
Prevalence of specific diseases for each of the ten categories of NMDs. **A**: Acquired peripheral neuropathy; **B**: genetic peripheral neuropathy; **C**: inflammatory myopathy; **D**: metabolic myopathy; **E**: nondystrophic myopathy; **F**: muscular dystrophy; **G**: motor neuron disease; **H**: neuromuscular junction disease; **I**: muscular channelopathy; **J**: hereditary ataxia and spastic paraplegia

The prevalence of specific pathologies within the ten NMD categories for confirmed diagnosis (n = 891) is illustrated in Fig. 2**.** A major proportion of acquired neuropathies were of autoimmune etiology, which included CIDP (n = 105) and multifocal motor neuropathies (MMN) (n = 18). The most frequent genetic neuropathies were Charcot-Marie-Tooth neuropathies (CMT) (n = 134), especially CMT1A (n = 89), which is associated with the duplication of the *PMP22* gene or a mutation in this gene.

For myopathies, autoimmune causes were the most common cause among nondystrophic myopathies, mostly inflammatory myopathies (n = 65). Congenital and metabolic myopathies (n = 13 and 14, respectively) were less frequent.

The largest proportion of muscular dystrophies was muscular dystrophy (MD) type 1, which occurred in 96 patients. Approximately two-thirds of the patients who carried *DMD* gene missense variants or deletions had Duchenne muscular dystrophies (DMD), one-third had Becker muscular dystrophies (BMDs), and two were female symptomatic carriers. The third and fourth most common dystrophies were FSHD (n = 41) and limb girdle muscular dystrophies (LGMD) (n = 23).

Motor neuron diseases (MND) in adults were mostly ALS, with 36 patients currently being followed. In children, SMA is the most common MND. Twelve of those pediatric SMA patients (24%) were not identified by symptoms (NIS), defined as having been diagnosed without symptoms at birth by neonatal screening or as being siblings of an identified patient [18]. The proportions of SMA types were 20% for type 1 (n = 10), 26% for type 2 (n = 13), and 30% for type 3 (n = 15). Only one patient had a mutation in the *SMN1* gene associated with a compound deletion of exon 7. All the others had homozygous deletions of at least exon 7.

Neuromuscular junction diseases (NMJD) were essentially characterized by myasthenia, mainly of autoimmune origin (n = 29), rather than congenital myasthenic syndromes (n = 7).

The most common channelopathy was myotonia congenita, which was associated with variants in the *CLCN1* gene in 10 of the 11 patients, and one was associated with a variant in the *SCN4A* gene.

Among patients suffering from ataxia (n = 13), 5 patients had Friedreich ataxia associated with homozygous expansion in the *FXN* gene, while 2 patients had a single nucleotide pathogenic variant in this gene and a compound heterozygous expansion. The most widely represented hereditary spastic paraplegia (HSP) was autosomal dominant spastic paraplegia type 4 (SPG4), which is associated with mutations in the *SPAST* gene (n = 8). The prevalence of specific diseases for each of the ten categories of NMD is available in Supplementary Tables 1–10.

### Diagnostic process

**Fig. 3:**
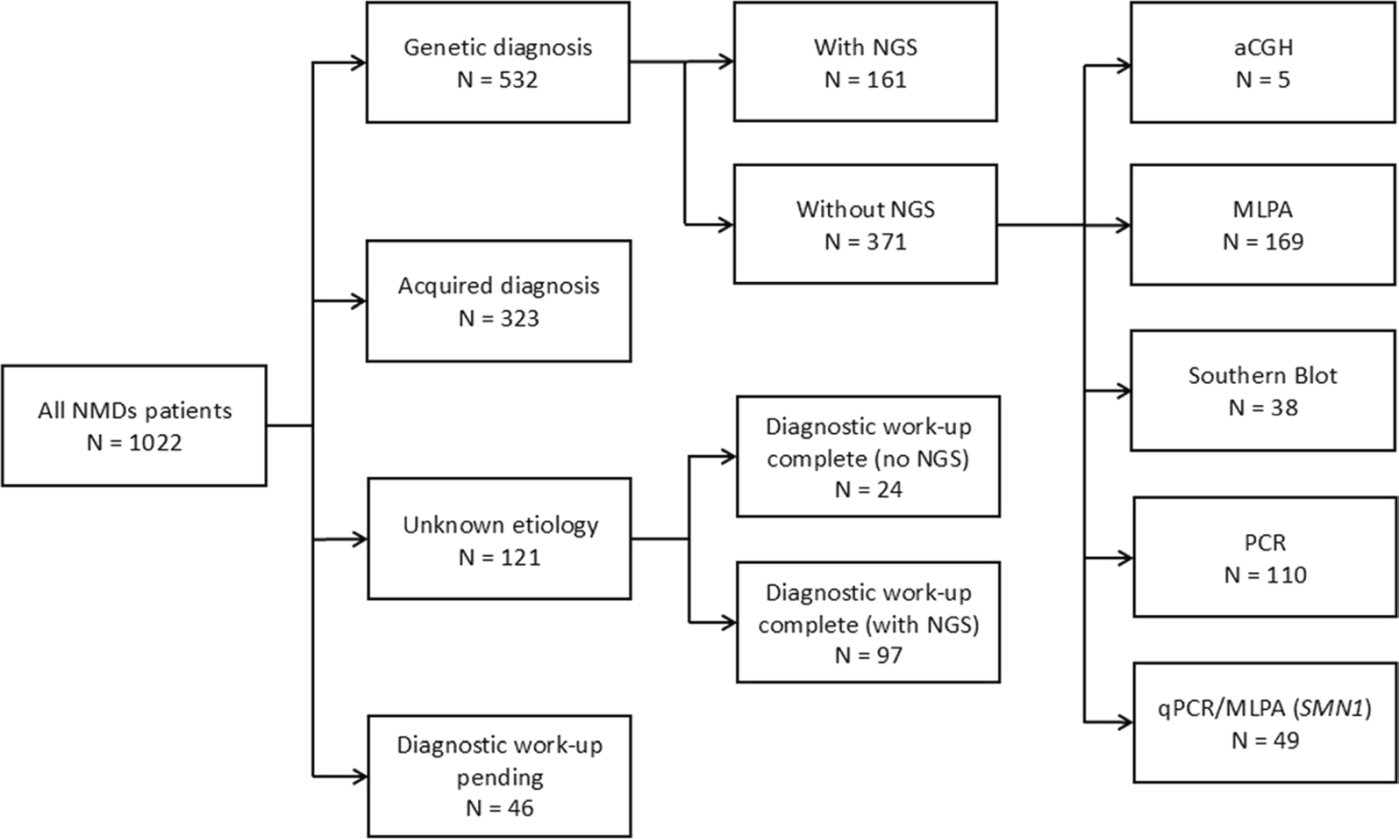
Distribution of acquired, genetic, and unknown diagnoses depending on NGS use

Of the 1084 patients, 889 had a definite diagnosis with 324 acquired etiologies and 565 genetic etiologies (Fig. 3). Among genetically confirmed cases, 32.7% were diagnosed by NGS, primarily through NGS gene panels with a few WES analyses and no WGS, due to limited access as previously described. The other genetic tests that led to diagnosis accounted for 67.3%. Five diagnoses were made using aCGH including two duplications of *PMP22*, two deletions of the *DMD* gene and a microdeletion syndrome. MLPA was used for *DMD* and *PMP22* analysis and represented 168 diagnoses. Forty-nine deletions of exon 7 of *SMN1* were detected by either MLPA (N = 25) or (qPCR) (N = 24). qPCR was used for newborn screening, and MLPA was used for symptomatic patients. The contraction of the D4Z4 microsatellite repeat in the 4q35 region associated with FSHD1 was assessed by southern blot (n = 38). Finally, repeat expansion diseases were confirmed by PCR. With this technique we found nine ataxias, including five Friedreich ataxias and one cerebellar ataxia, neuropathy, and vestibular syndrome (CANVAS), 96 MD type 1, nine MD type 2, three spinal and bulbar muscular atrophies (SMAX1), one ALS associated with expansion in *C9Orf72*, and two oculopharyngeal muscular dystrophies (OPMD1).

The total percentage of undiagnosed patients was 13.7%. The rates for all the NMD categories are illustrated in Supplementary Table 11. For neuropathies, 13.9% had acquired etiologies, and 25.1% had genetic etiologies. High percentages of patients had myopathies, especially nondystrophic myopathies, and metabolic myopathies (53.5% and 48.2%, respectively). The uncertainty was lower for inflammatory myopathies (11.0%) and muscular dystrophies (12.1%). The lowest number of unsolved cases was found in motor neuron diseases (3.3%), followed by NMJD (7.5%) and channelopathies (8.7%). Overall, there was a high unconfirmed diagnostic rate of 43.5% for hereditary ataxia and spastic paraplegia. The diagnostic rate was largely similar between adult and pediatric populations, with the exception of neuropathies—particularly acquired forms—which showed a significantly higher rate of unconfirmed diagnoses in adults. In contrast, congenital myopathies were more frequently unconfirmed in children. No cases of inflammatory myopathy were observed in the pediatric population.

## Discussion

### Neuropathies

This study illustrates the prevalence of NMD in the NMRC of Liege and the diagnostic approach used in this cohort. We chose to focus on the three main subgroups of NMD (i.e., neuropathies, myopathies, and motor neuron diseases) in order to compare our data with current diagnostic guidelines. This approach is applicable to other rarer categories.

**Fig. 4:**
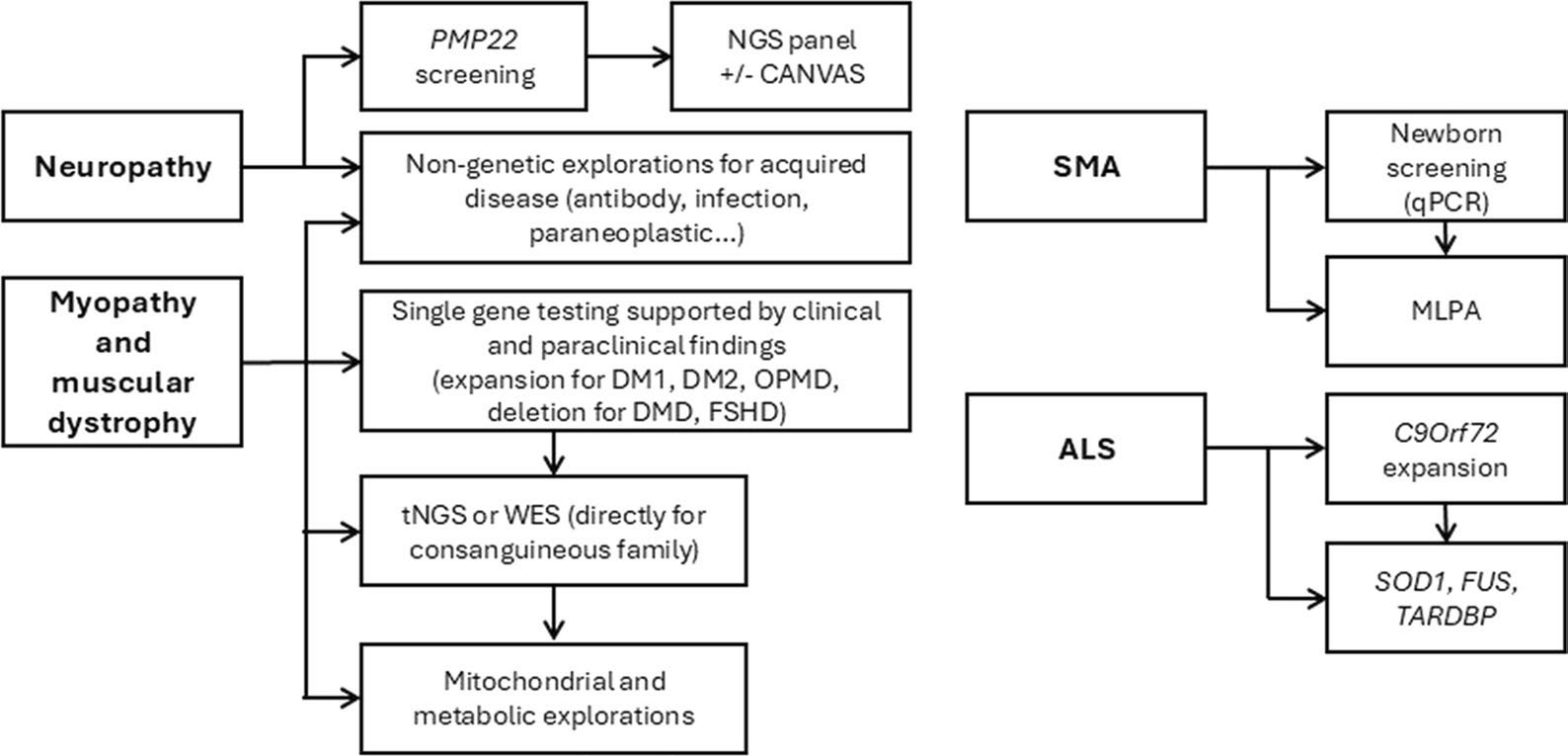
Proposition of diagnostic guidelines for the most frequent NMDs (i.e., neuropathies, myopathies, and motor neuron diseases)

Our results show that the most common pathology followed up in our center is neuropathy, which affects almost half of the patients. The most frequent neuropathy is CIDP. The number of CIDP patients (11.8%) may seem high compared to other studies that reported 3 to 6.7% of their NMD cohort [2, 19]. However, two factors could explain this difference. First, in Belgium, unlike other neuropathies, CIDP patients must be followed in the NMRC to benefit from IVIg, and the center of Liege is the only NMRC in the southern part of Belgium. Furthermore, we currently use the EAN/PNS criteria, which are more sensitive than those developed by the Ad Hoc Subcommittee of the American Academy of Neurology (AAN) [20]. The prevalence of genetically confirmed neuropathies (16.4%) was also greater than that previously reported in other centers [19, 21]. This could be explained by the use of a comprehensive NGS panel. However, even after a complete work-up including NGS, 19.4% of patients were undiagnosed, which is in line with the literature describing 20 to 30% of idiopathic neuropathies [22]. A considerable proportion of these neuropathies is likely genetic. For instance, CANVAS appears to be a frequent cause of neuropathy, with a growing number of diagnoses since the detection of *RCF1* gene repeat expansion [23]. Therefore, genetic testing should be part of the work-up even if there is no family history. Actual genetic guidelines recommend testing *PMP22* for the classical phenotype of CMT1A (i.e., hereditary demyelinating neuropathy) and proceeding to WGS for negative analysis and for other indications [24]. However, the CMT1A phenotype can be broad and has even been described as being associated with axonal neuropathy [25]. Second, WGS is not routinely available worldwide, and its interpretation remains complex. Therefore, a reasonable approach would be to screen for *PMP22* deletions and duplications prior to perform NGS testing [14]. Actually, such CNV in the *PMP22* gene are currently poorly covered by NGS. Subsequently, a comprehensive gene panel or WES should be performed. WES offers better efficacy if available [26] because it covers other genes associated with differential diagnosis [14]. *RCF1* expansion testing should also be considered, particularly when neuropathy is associated with ataxia, vestibular areflexia, or chronic cough [23] (Fig. 4**)**.

### Myopathies, including muscular dystrophies

The second most common group of pathologies in our center is myopathies, including muscular dystrophies. Genetically confirmed nondystrophic myopathies and muscular dystrophies account for approximately 30% of our population, with 10% of MD type 1 as reported in other NMRC [19, 21, 27, 28]. We would expect more genetically confirmed metabolic myopathy cases of approximately 1.5% compared to the 2.5–5% previously described in other centers [2, 19, 29]. A possible explanation could be a lack of or a delay in molecular confirmation, as it has been described for late-onset Pompe disease, for which the diagnostic delay is approximately 13 years in Belgium compared to 3–7 years in other countries [30]. Indeed, if unconfirmed patients are included, the percentage of that subgroup increases to 2.5%.

The main difficulty in the genetic diagnosis of myopathies is the large number of different types of causative variants, even in the same gene (e.g., deletions or missense variants in the *DMD* gene), for which a large number of techniques are required for detection and interpretation [1]. This genetic heterogeneity is also associated with phenotypic variability, even within the same family. This is likely responsible for our significant percentage of undiagnosed patients (i.e., approximately 50% for nondystrophic myopathies and metabolic myopathies). The rate is better for muscular dystrophies (12.1%). This higher diagnostic rate is likely attributable to the substantial proportion of patients with DMD-related disorders (e.g., DMD, BMD, and symptomatic female carriers), which represent over 7% of the muscular dystrophies. In these cases, systematic genetic testing is routinely performed due to the clinical importance of early and accurate diagnosis. Indeed, the emergence of disease-modifying therapies—such as exon-skipping treatments for Duchenne muscular dystrophy [31]—has reinforced the need for timely and comprehensive genetic screening. These therapeutic advances are driving a shift toward more systematic and proactive genetic testing strategies in clinical practice [31]. Genetic testing guidelines for muscular dystrophies and other myopathies are broad and do not necessarily consider phenotypic variability. The first step is single-gene testing of non-missense variants, such as small deletions (e.g., *DMD* [32]), large deletions (e.g., the 4qter region associated with FSHD 1 [33]) or expansions (e.g., *DMPK* and *CNBP*), based on clinical suspicion (Fig. 4). For other myopathies or following initial screening, current recommendations suggest performing NGS, either through targeted gene panels or WES [15]. Recent guidelines increasingly support WGS as the most comprehensive and high-performing approach [11]. However, beyond the current limited access to WGS, sequencing can also reveal multiple variants of unknown significance (VUS), particularly in large muscular genes. This is notably the case for genes such as *TTN*, which encodes a protein of 26,926 amino acids [34], and *DMD*, with 3,685 amino acids [35] leading to a high rate of VUS, making interpretation particularly challenging [36]. To support pathogenicity assessment, clinical and para-clinical investigations (e.g., EMG, imagery, muscular biopsy) can provide valuable evidence pointing toward a specific disease. However, while such evidence can support the diagnostic process, it may also be conflicting or point to overlapping genetic etiologies. For example, the pattern of muscle involvement on MRI in dysferlinopathies can appear similar to other myopathies [37], and there is significant phenotypic overlap between congenital myopathy genes and muscular dystrophy genes, complicating interpretation [15]. The complexity of variant interpretation can be improved by preanalytical reflection and discussion during multidisciplinary meetings. Phenotyping (i.e., clinical and paraclinical examination) should still drive the choice of genetic analysis, especially for disorders not associated with single-nucleotide variants (SNV) (e.g., deletions and expansions), which are currently poorly detected by NGS (Fig. 4). Moreover, NGS panels appear to be sufficient compared to WES except for consanguineous families in which a rarer recessive disease is suspected [38]. In addition, mitochondrial analysis, which should include at least a NGS panel targeting mitochondrial nuclear genes and the mitochondrial genome, should be performed when there is a suspicion of a mitochondriopathy [39]. In the case of exercise intolerance, even in adulthood, metabolic screening, especially for Pompe disease, should be sought since it may present as isolated exercise intolerance and replacement therapy is available [40].

### Motor neuron diseases

The third most frequent disorder in our cohort was MND, mainly represented by SMA and ALS. SMA accounts for 50 patients, which is less common than expected [29, 41] since the incidence of SMA in Belgium is estimated to be approximately 1:14,000 [18]. However, in the pediatric population, our data (37.1%) are in line with those of other centers [29]. Today, in Belgium, all newly diagnosed SMA patients are followed up in an NMRC for access to treatments such as nusinersen (Spinraza^®^), onasemnogene abeparvovec (Zolgensma^®^), or risdiplam (Evrysdi^®^). In addition, a newborn screening program, which has been in place since 2018 in Wallonia, diagnoses patients at birth, most often at a presymptomatic stage [18]. However, before the advent of disease-modifying drugs and newborn screening, a considerable proportion of patients died during childhood, approximately 18 months for type 1 disease and before the age of 18 years for type 2 disease [42]. These findings explain the greater prevalence of SMA type 1 compared to other reports conducted in countries without newborn screening or with poor access to disease-modifying drugs [21, 29]. These data should therefore evolve over time. The same trend toward better diagnosis is applicable for ALS because of promising disease-modifying drugs [43]. The low proportion of undiagnosed patients highlights the impact of new therapies on diagnostic methods. Given the availability of disease-modifying drugs, the diagnostic journey of SMA should be well established with neonatal screening [44] as well as screening for *C9Orf72* expansion and at least *SOD1, FUS,* and *TARDBP* SNV for all new ALS patients [45, 46] (Fig. 4). NGS panels or WES can be performed in cases of familial ALS. However, regardless of age at onset or family history, testing for *SOD1* and *FUS* variants should be considered, as promising targeted therapies are currently emerging for these specific genes [47].

### Implementation of NGS in NMD diagnosis

Of over 500 genetic diagnoses, only approximately one-third have been resolved using NGS, mainly targeted gene panels. Only a few patients underwent WES and no WGS was performed due to limited access through research programs, prohibitive cost and the complexity of data analysis. This finding illustrates the importance of alternative genetic methods, such as MLPA, PCR, and aCGH. To date, deletions, duplications, or expansions are still poorly detected by short-read sequencing and constitute a common pathological mechanism of NMD [1]. The diagnostic yield depends on the sensitivity of the technique. Actual gene panels and WES focus on SNV or small insertions/deletions (indels) because short-read DNA sequencing technologies are best suited for identifying these variants. However, these methods are less sensitive to the identification of CNV and may miss large deletions or duplications. To overcome this limitation, strategies have been developed to call CNV from exome data with algorithms using read-depth data (e.g., ExomeDepth) [48]. In addition to new types of sequencing, called third-generation sequencing or long read sequencing, are used to detect CNV [49]. Long-read WGS will potentially be the future of DNA sequencing since it allows the detection of all types of variants (i.e., SNV, CNV, and expansion). However, this involves a considerable number of challenges to enable its worldwide use in terms of cost and infrastructure, but also for variant interpretation. The health care system should remain aware of these developments to adapt to resource allocations accordingly.

## Conclusion

This study showed that NMD are complex neurological disorders characterized by phenotypic overlap between subgroups and nonspecific clinical presentation associated with numerous acquired and genetic etiologies. Extensive diagnostic guidelines have been published to date, but they are not fully applicable in clinical practice due to cost, availability, and complexity of results. Long-read WGS will probably replace NGS panels and other traditional techniques (e.g., MLPA, PCR, aCGH) in the future. However, due to their lower cost and the challenges associated with variant interpretation in more advances techniques, these older techniques should still be used in clinical practice. This is supported by the diagnostic algorithm we propose (Fig. 4).

## Supplementary Information

Additional file1 (DOCX 113 KB)

## Data Availability

Pseudoanonymous patient data cannot be shared with nonmembers of the NMRC of the Liege team, as described in the ethical committee statement. De-identified, aggregated dataset summarizing the results could be made available to researchers upon reasonable request to enhance transparency and reproducibility.
