# Supplementary material for: Epidemiological report and diagnostic approach used in the neuromuscular population of Liege, Belgium
Source: Orphanet J Rare Dis. 2025 Aug 29;20:464. doi: 10.1186/s13023-025-03963-2 (PMC12398174; doi:10.1186/s13023-025-03963-2)
Supplement: Supplementary file 1 — Additional file1 (DOCX 113 KB) [file 13023_2025_3963_MOESM1_ESM.docx]

**Supplementary Material: Neuromuscular diseases prevalence and undiagnostic rate in Neuromuscular Reference Center of Liege, Belgium.**

**Supplementary Table 1. Acquired neuropathies prevalence**

| **Acquired polyneuropathy** | **Total of confirmed adult patients (N = 169)** | | **Total of confirmed pediatric patients (N = 5)** | |
| --- | --- | --- | --- | --- |
| Acquired amyloidosis | 1 | 0 | |  |
| Acquired sensory ganglionopathy | 1 | 0 | |  |
| CIDP | 104 | 1 | |  |
| Guillain-Barré syndrome | 11 | 2 | |  |
| Infectious disease with neuropathy | 0 | 1 | |  |
| MNM | 18 | 0 | |  |
| Other acquired peripheral neuropathy | 5 | 0 | |  |
| Parsonage Turner | 1 | 0 | |  |
| POEMS syndrome | 1 | 0 | |  |
| Polyneuropathy associated with IgM gammapathy and anti-MAG | 6 | 0 | |  |
| Systemic inflammatory disease associated with polyneuropathy | 9 | 0 | |  |
| Toxic and iatrogenic polyneuropathy | 12 | 1 | |  |

CIDP: Chronic inflammatory demyelinating polyneuropathy; MNM: Multifocal motor neuropathy; POEMS: Polyneuropathy, organomegaly, endocrinopathy, monoclonal gammopathy and skin abnormalities.

**Supplementary Table 2. Genetic neuropathies prevalence**

| **Genetic polyneuropathy** | **Total of confirmed adult patients (N = 120)** | **Total of confirmed pediatric patients (N = 26)** |
| --- | --- | --- |
| Adrenomyeloneuropathy | 1 | 0 |
| Axonal CMT | 10 | 3 |
| CMT1A | 72 | 17 |
| Demyelinating CMT | 11 | 4 |
| HNPP | 14 | 0 |
| Metachromatic leucodystrophy | 1 | 0 |
| Other genetic peripheral neuropathy | 0 | 2 |
| TTR-FAP | 8 | 0 |
| X-linked CMT | 3 | 0 |

CMT: Charcot-Marie-Tooth disease ; HNPP: Hereditary neuropathy with pressure palsies ; TTR-FAP: Transtiretin-related familial amyloid neuropathy.

**Supplementary Table 3. Inflammatory myopathies prevalence**

| **Inflammatory myopathy** | **Total of confirmed adult patients (N = 65)** | **Total of confirmed pediatric patients (N = 0)** |
| --- | --- | --- |
| Antisynthetase syndrome | 13 | 0 |
| Dermatopolymyositis | 17 | 0 |
| Immune-mediated necrotizing myopathy | 10 | 0 |
| Inclusion body myopathy | 9 | 0 |
| Other inflammatory myopathy | 8 | 0 |
| Overlap myositis | 5 | 0 |
| Polymyositis | 3 | 0 |

**Supplementary Table 4. Metabolic myopathies prevalence**

| **Metabolic myopathy** | **Total of confirmed adults patients (N = 12)** | **Total of confirmed pediatric patients (N = 2)** |
| --- | --- | --- |
| McArdle disease | 3 | 1 |
| Mitochondrial myopathy | 3 | 0 |
| Muscular lipidosis | 1 | 1 |
| Other metabolic myopathy | 3 | 0 |
| Pompe disease | 2 | 0 |

**Supplementary Table 5. Non-dystrophic myopathies prevalence**

| **Non-dystrophic myopathy** | **Total of confirmed adult patients (N = 17)** | **Total of confirmed pediatric patients (N = 3)** |
| --- | --- | --- |
| Congenital myopathy | 10 | 3 |
| Distal myopathy | 3 | 0 |
| Inclusion myopathy | 1 | 0 |
| Myofibrillar myopathy | 1 | 0 |
| Other non-dystrophic myopathy | 2 | 0 |

**Supplementary Table 6. Muscular dystrophies prevalence**

| **Muscular dystrophy** | **Total of confirmed adult patients (N = 211)** | **Total of confirmed pediatric patients (N = 51)** |
| --- | --- | --- |
| Becker muscular dystrophy | 16 | 7 |
| Congenital muscular dystrophy | 6 | 3 |
| Duchenne muscular dystrophy | 19 | 30 |
| Duchenne symptomatic carrier | 6 | 1 |
| Emery-Dreifuss muscular dystrophy | 1 | 0 |
| Facioscapulohumeral muscular dystrophy | 41 | 0 |
| Limb Girdle muscular dystrophy | 22 | 1 |
| Oculopharyngeal muscular dystrophy | 2 | 0 |
| Other muscular dystrophy | 1 | 1 |
| PROMM | 7 | 2 |
| Steinert muscular dystrophy | 90 | 6 |

PROMM: proximal myotonic myopathy.

**Supplementary Table 7. Motor neuron diseases prevalence**

| **Motor neuron disease** | **Total of confirmed adult patients (N = 83)** | **Total of confirmed pediatric patients (N = 34)** |
| --- | --- | --- |
| Amyotrophic lateral sclerosis | 36 | 0 |
| Bulbospinal muscular atrophy | 3 | 0 |
| Poliomyelitis sequalae | 25 | 0 |
| Proximal spinal muscular atrophy | 2 | 1 |
| SMA not identify by symptoms | 0 | 12 |
| SMA type 1 | 0 | 10 |
| SMA type 2 | 6 | 7 |
| SMA type 3 | 11 | 4 |

SMA: Spinal muscular atrophy.

**Supplementary Table 8. Neuromuscular junction diseases prevalence**

| **Neuromuscular junction disease** | **Total of confirmed adult patients (N = 31)** | **Total of confirmed pediatric patients (N = 6)** |
| --- | --- | --- |
| Congenital myasthenic syndrome | 4 | 3 |
| Lambert-Eaton myasthenic syndrome | 1 | 0 |
| Myasthenia gravis | 26 | 3 |

**Supplementary Table 9. Muscular channelopathies prevalence**

| **Muscular channelopathy** | **Total of confirmed adult patients (N = 18)** | **Total of confirmed pediatric patients (N = 3)** |
| --- | --- | --- |
| Genetic periodic paralysis | 2 | 0 |
| Myotonia congenita | 9 | 3 |
| Other muscular channelopathy | 3 | 0 |
| Paramyotonia congenita | 4 | 0 |

**Supplementary Table 10. Hereditary ataxias and spastic paraplegias prevalence**

| **Hereditary ataxia and spastic paraplegia** | **Total of confirmed adult patients (N = 25)** | **Total of confirmed pediatric patients (N =10 )** |
| --- | --- | --- |
| Autosomal dominant cerebellar ataxia | 4 | 1 |
| Autosomal recessive cerebellar ataxia | 2 | 0 |
| Friedreich ataxia | 4 | 1 |
| Hereditary spastic paraplegia | 14 | 8 |
| Other ataxia | 1 | 0 |

**Supplementary table 11. Unconfirmed diagnostic rate for each NMDs category**

| **Neuromuscular disorder** | **Unconfirmed diagnostic rate for adult patients  (N = 911)** | **Unconfirmed diagnostic rate for pediatric patients (N = 173)** | **Total unconfirmed diagnostic rate  (N = 1084)** |
| --- | --- | --- | --- |
| Acquired peripheral neuropathy | 28/197 (14.2 %) | 0/5 (0.0 %) | 174/202 (13.9 %) |
| Genetic peripheral neuropathy | 43/163 (26.4 %) | 6/32 (18.8 %) | 146/195 (25.1 %) |
| Inflammatory myopathy | 8/73 (11.0 %) | 0/0 (NA) | 65/73 (11.0 %) |
| Metabolic myopathy | 12/24 (50.0 %) | 1/3 (33.3 %) | 14/27 (48.2 %) |
| Non-dystrophic myopathy | 15/32 (46.9 %) | 8/11 (72.7 %) | 20/43 (53.5 %) |
| Muscular dystrophy | 29/240 (12.1 %) | 7/58 (12.1 %) | 262/298 (12.1 %) |
| Motor neuron disease | 2/85 (2.4 %) | 2/36 (5.6 %) | 117/121 (3.3 %) |
| Neuromuscular junction disease | 2/33 (6.1 %) | 1/7 (14.3 %) | 37/40 (7.5 %) |
| Muscular channelopathy | 2/20 (10.0 %) | 0/3 (0 %) | 21/23 (8.7 %) |
| Hereditary ataxia and spastic paraplegia | 19/44 (46.9 %) | 8/18 (44.4 %) | 35/62 (43.6 %) |
